# Improving Multivariate Time-Series Anomaly Detection in Industrial Sensor Networks Using Entropy-Based Feature Aggregation

**DOI:** 10.3390/e28010014

**Published:** 2025-12-23

**Authors:** Bowen Wang

**Affiliations:** School of Electronics and Information Engineering, Beihang University, Beijing 100191, China; wangbowen213@buaa.edu.cn

**Keywords:** anomaly detection, industrial sensor networks, graph neural networks, structural entropy

## Abstract

Anomaly detection using multivariate time-series data remains a significant challenge for complex industrial systems, such as Cyber–Physical Systems (CPSs), Industrial Control Systems (ICSs), Intrusion Detection Systems (IDSs), the Internet of Things (IoT), and Remote Sensing Monitoring Platforms, including satellite Earth observation systems and Mars Rovers. In these systems, sensors are highly interconnected, and local anomalies frequently affect multiple components. Because these interconnections are often implicit and involve complex interactions, systematic characterization is required. To address this, our study employs graph neural networks with a structure-entropy-based attention mechanism, which models multi-element relationships and formally represents implicit relationships within complex industrial systems using a network-based structural model. Specifically, our method distinguishes the weights of different high-order neighbor nodes based on their locations, rather than treating all nodes equally. Through this formalization, we identify and represent key adjacent elements by analyzing system entropy. We validate our method on SMAT, MSL, SWaT, and WADI datasets, and experimental results demonstrate improved detection performance compared to baseline approaches.

## 1. Introduction

**Figure 1:**
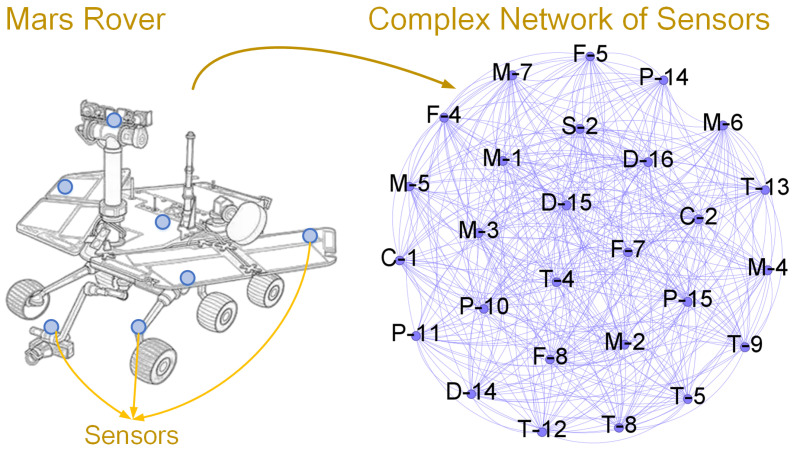
The 27 sensors on the Mars Rover record time-series signals, including cameras, spectrometers, and environmental sensors. All sensors construct a complex network with edges indicating potential connections.

Anomaly detection [1,2] is a critical interdisciplinary domain focused on identifying deviations from standard operational patterns within complex industrial environments. This capability is essential across various industrial systems engineering fields, such as Cyber–Physical Systems (CPSs) [3], Industrial Control Systems (ICSs) [4], Intrusion Detection Systems (IDSs) [5], the Internet of Things (IoT) [6], and Remote Sensing Monitoring Platforms (e.g., satellite Earth observation systems and Mars Rovers). The core goal of anomaly detection is to identify potential faults in these industrial systems, which are usually evidenced by the oscillation of time-series signals collected by multiple sensors throughout the system. For example, in the NASA Mars Science Laboratory (MSL) [7] (shown in Figure 1), multiple measurements from cameras, spectrometers, and environmental sensors record time-series signals. Anomalous sensor data in the MSL system can disrupt operational logic, indicating possible malfunctions of the Mars Rover.

Current industrial anomaly detection faces three primary challenges that require the attention of researchers. The first challenge is the inherent complexity of industrial systems, which consist of numerous interconnected components such as machinery, sensors, control systems, and human–machine interfaces. These components interact non-linearly, resulting in systems that exhibit dynamic and evolving behaviors [8,9,10]. The second challenge involves the multifactorial origin of faults. Although diverse time series can be used to characterize the temporal evolution of each element (sensor) separately for detection and analysis, neglecting the system’s architecture risks disregarding the impact of physical and logical coupling among variables on fault detection. The third challenge is the stability of industrial system sensors, which usually remain stable for extended periods, changing only during specific time intervals. These shifts can alter overall system properties, potentially leading to faults. This phenomenon suggests that a minor alteration in a critical parameter can induce a qualitative shift in the system’s state [11,12].

Recently, there has been increasing interest in structurally modelling various industrial systems using multivariate time series correlations. In such modelling, the significance of sensors is characterised by the structure’s importance, and structural changes show sensor variations over time. A particularly promising direction is graph modelling, which has gained attention for directly bridging the gap between complex industrial systems and various machine learning techniques. For instance, basic Graph Convolutional Networks (GCNs) [13] treat each node as a sensor and learn structural relationships among sensors through information aggregation and propagation. Building upon this, recent research has integrated time series modelling with GCNs. For example, GTAD [14] introduced an automated graph modelling approach for IoT that utilises a transformer architecture to capture the temporal characteristics of data. Similarly, MTGNN [15] employs a novel mix-hop propagation layer and a dilated inception layer to capture spatial and temporal dependencies within the time series. In addition, StemGNN [16] applies graph spectral theory to multivariate time series modelling, explicitly using the Graph Fourier Transform (GFT) [17] to model inter-series correlations and the Discrete Fourier Transform (DFT) [18] to model temporal dependencies within an end-to-end framework. Finally, LGAT [19] automatically learns graph structures and leverages an enhanced Anomaly Transformer architecture to capture temporal dependencies. Another strategy to tackle anomaly detection is the imputation-based method, which offers a straightforward solution for incomplete data by filling gaps. This strategy assumes that signals could be accurately modeled. Typical methods in this field are ImDiffusion [20], DiffAD [21], and DSDI [22].

Graph modeling theory and its associated methodologies provide innovative strategies for anomaly detection in complex industrial systems [23]. Building on these foundations, we employ graph models to extract and represent data from multiple sensors through a neural network architecture. We introduce an entropy-based attention mechanism to generate node representations within the network. For clarity, our approach is organized into four main sections. First, we describe the Graph Structure of Sensors, utilizing directed graphs to identify implicit relationships among sensors and selecting the top K related nodes for each sensor. Second, we present the Entropy Centrality-based Graph Attention, which integrates all modules within a graph neural network framework and incorporates an entropy-based attention module to aggregate information and represent each node. Third, we detail the Loss and Training process to explain how the model is trained and its parameters are updated using gradient descent. Finally, we perform anomaly detection, which calculates the mean deviation between predicted and expected values to identify anomalies.

The innovations of this paper correspond to the main modules, which are as follows:

- This work employs a graph model to reveal hidden structures in complex industrial systems by examining sensor interaction relationships.
- This work introduces Entropy Centrality-based Graph Attention, which screens each node’s neighbors by analyzing entropy decay strength in the graph’s structure.
- Experiments have shown that this method enhances the efficiency of anomaly detection tasks.

The rest of this paper is structured as follows. Section 2 introduces the definitions of multivariate time-series data and graph models. Section 3 introduces the framework of this work, including the Graph Structure of Sensors, Entropy Centrality-based Graph Attention, and anomaly detection. Section 4 presents various experiments, essential details, results, and discussions. Section 5 summarizes this work and provides a future outlook.

## 2. Problem Statement

### 2.1. Multivariate Time-Series Data

In this work, we focus on multivariate time-series anomaly detection. Let $x^{\left(t\right)}\in R^{N\times 1}$ denote the original multivariate time-series data at any time stamp *t*, where *N* is the total number of sensors and $x_{i}^{\left(t\right)}$ is the *i*-th sensor’s record data. The high imbalance between normal data and anomalies is a common characteristic of various anomaly detection tasks, where the amount of normal data is significantly greater than that of anomalies. Anomaly detection aims to predict anomalies in the test data based on normal data.

In mathematics, anomaly detection aims to learn a prediction function F that maps the input to the label field based on historical ω-step time series. Specifically, the *m*-th **input** is denoted as the ω-step time series

(1) $$\begin{array}{cc} X^{m} & :=[X_{1}^{m},\ldots ,X_{N}^{m}]\\ \; & =[x^{\left(m\omega +(m-1)\tau \right)},\ldots ,x^{\left((m-1)\omega +(m-1)\tau \right)}]. \end{array}$$

where τ is the **sliding step**. Here, $X_{i}^{m}$ is the *i*-th sensor’s ω-step record time series. This prediction function is denoted as

(2) $$F:X^{m}\to Y,X^{m}\mapsto Y,$$

where *Y* is the **binary label** pending prediction and ω is the **window size**. Then, the training dataset collects a set of *M* different inputs:

(3) $$X^{Train}=\left\{X^{1},\ldots ,X^{M}\right\}.$$

**Figure 2:**
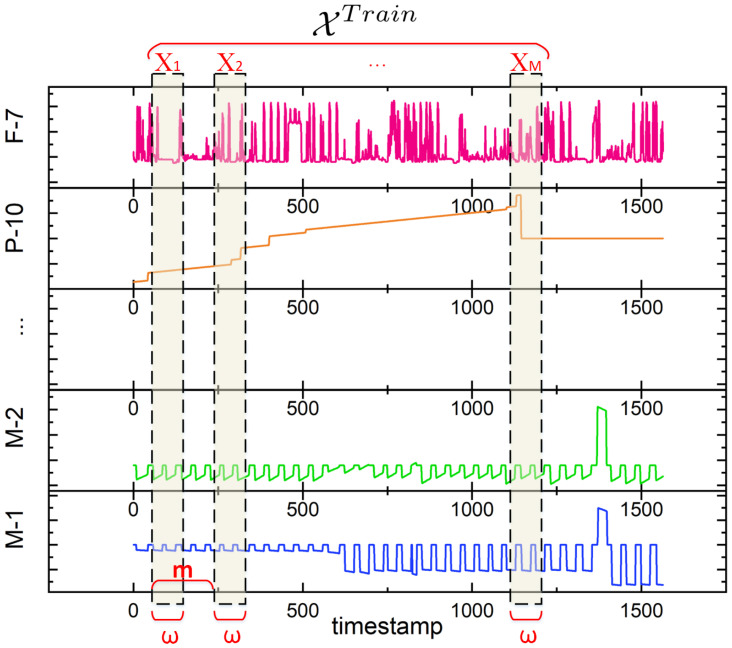
The training data for the work. Each training dataset is an ω-length multivariate time series with a sliding step of τ.

The visualization of the training set is presented in Figure 2.

### 2.2. Graph Model

Graph is a kind of structural data that organizes various items within a system with edges connecting them. Formally, a graph is denoted as G={V,E}, where $V=\{v_{1},\ldots ,v_{n}\}$ consists of *n* elements and E⊆V×V is the edge set.

Graphs can be categorized as undirected or directed based on the directionality of their edges. Undirected graphs have edges without directionality, meaning that $\left(v_{i},v_{j}\right)=\left(v_{j},v_{i}\right)$. Directed graphs have edges with explicit directionality, implying that $\left(v_{i},v_{j}\right)\neq \left(v_{j},v_{i}\right)$. Specifically, for an edge $\left(v_{i},v_{j}\right)$ with direction $v_{i}\to v_{j}$, the node $v_{i}$ refers to the source node and $v_{j}$ is the target node. The directionality of graph edges is typically captured using an n×n adjacency matrix A, where the matrix entry at row *i* and column *j* indicates the presence or absence of an edge from node $v_{i}$ to node $v_{j}$. A is determined as follows:

(4) $$A_{ij}=\left\{\begin{array}{cc} 1, & if\;(v_{i},v_{j})\in E,\\ 0, & otherwise. \end{array}\right.$$

## 3. Method

**Figure 3:**
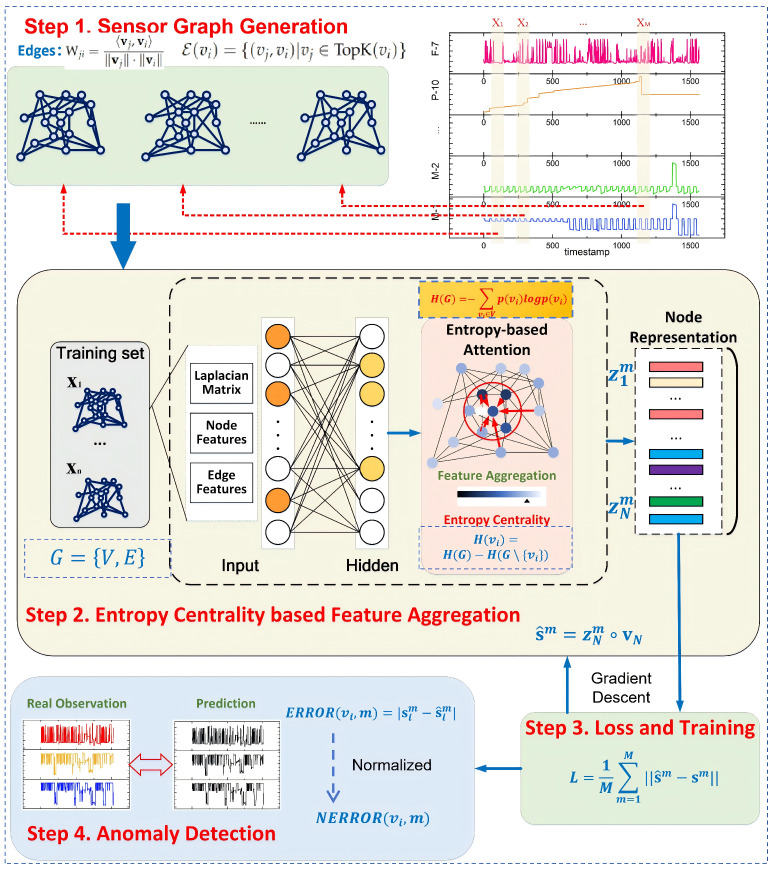
The proposed method comprises four main steps: (1) Sensor Graph Generation uses directed graphs to find hidden links among sensors in industrial systems, selecting the top *K* related nodes for each node. (2) Entropy Centrality-Based Feature Aggregation puts all modules into a graph neural network and uses an Entropy-Based Attention module to combine information and represent each node. (3) Loss and Training trains and updates parameters using gradient descent. (4) Anomaly detection finds anomalies by measuring the mean difference between predicted and expected values.

The method includes three parts: Graph Structure of Sensors, Entropy Centrality-based Graph Attention, and anomaly detection. The framework is shown in Figure 3.

- **Step 1. Sensor Graph Generation** utilizes directed graphs to extract implicit relationships among sensors in complex industrial systems, selecting the top K related nodes for each node.
- **Step 2. Entropy Centrality-based Feature Aggregation** integrates all preceding modules within a graph neural network framework by employing an entropy-based attention module that gathers information from related nodes and represents each node accordingly.
- **Step 3. Loss and Training** trains and updates parameters vis gradient descent.
- **Step 4. Anomaly Detection** implements anomaly detection by calculating the mean deviation between predicted and expected values.

### 3.1. Sensor Graph Generation

This section aims to model the graph structure of complex industrial systems based on multivariate time-series data. It primarily involves representing system participants as nodes and modeling the relationships between these participants within industrial systems. Specifically, we denote $G_{m}$ as the Sensor Graph derived from *m*-th ω-length multivariate time-series $X^{m}$.

#### 3.1.1. Representing Nodes

In complex industrial systems, including the NASA Mars Science Laboratory (MSL) rover dataset, data is collected from multiple sensors monitoring various rover components. When a fault occurs, several sensors often trigger alerts at once, indicating correlations among specific sensors. These correlations are difficult to spot in multivariate time-series data. This section aims to represent sensors as graph nodes using learnable vectors. These vectors are then optimized by model training in later sections. In this context, we employ the graph model G=(V,E) to depict a sensor network. The set $V=\{v_{1},\ldots ,v_{n}\}$ represents the nodes, and the set $E=\{\left(v_{1},v_{2}\right),\ldots ,\left(v_{n-1},v_{n}\right)\}$ represents the connections between them. To obtain a vector representation for each node, we utilize learnable vectors encapsulated in an n×d feature matrix V. Each row $v_{i}\in R^{d}$ within this matrix corresponds to the feature vector for node $v_{i}$ in the *m*-th graph, $G^{m}$, where *i* ranges from 1 to *n*.

#### 3.1.2. Representing Edges

To elucidate the interdependencies within complex industrial systems, this paper employs directed edges and graphs to model the influence exerted by one sensor upon others. Since structural relationships among sensors are generally absent, the most straightforward approach is to construct a fully connected graph, in which each sensor is influenced by all others. However, this representation is overly coarse. Therefore, this paper focuses solely on the top K sensors that significantly influence a given sensor.

For a node $v_{i}\in G$ and any other node $v_{j}\in G$, the weight of any possible directed edge $\left(v_{j},v_{i}\right)$ can be represented by a normalization function:

(5) $$W_{ji}=\frac{\langle v_{j},v_{i}\rangle }{∥v_{j}∥\cdot ∥v_{i}∥}.$$

Then, the real edges are selected via arranging the top-k weight scores of all possible edges connected to $v_{i}$. That is,

(6) $$E\left(v_{i}\right)=\left\{\left(v_{j},v_{i}\right)|v_{j}\in \mathrm{TopK}\left(v_{i}\right)\right\},$$

where $\mathrm{TopK}(v_{i})$ is the set of nodes that derives the top *K* weighted scores. Consequently, the directed edge structure of the graph can be obtained and represented by the directed adjacency matrix A, where $A_{ji}=1$ if $v_{j}\in E\left(v_{i}\right)$.

### 3.2. Entropy Centrality-Based Feature Aggregation

In industrial systems, components and sensors construct complex networks through the Industrial Internet and the Industrial Internet of Things. Each sensor acts as a node within the system’s overall graph structure, while the connections between them represent system interactions. This complex system builds upon the previously described graph model, which captures its topological features. Because structure embodies information, this section will assess the significance of each node (each sensor) within the entire graph (the industrial complex system) from an information entropy perspective.

**Figure 4:**
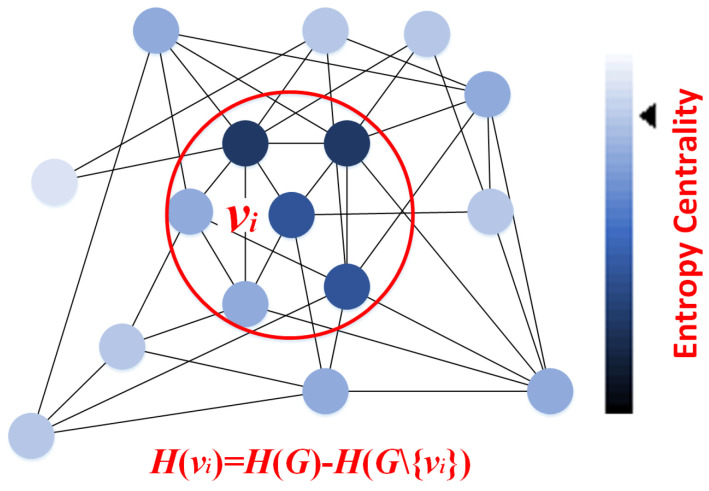
The entropy-based centrality for each node $v_{i}$ within graph *G*, which is $H\left(v_{i}\right)=H\left(G\right)-H\left(G\setminus \{v_{i}\}\right)$.

Entropy is a fundamental concept in statistical physics, and the second law of thermodynamics indicates that it is challenging to decrease the entropy of a macroscopic system [24]. Shannon introduced the idea of entropy into information theory as a measure to quantify the information associated with a system [25,26]. A key conclusion is that the more essential an element is, the greater its contribution to the system’s entropy [27]. Therefore, removing a critical element results in a more significant decrease in the system’s overall entropy (Figure 4).

For a discrete system S and each element s∈S, the basic definition entropy of S is denoted as

(7) $$H\left(S\right)=-\sum_{s\in S}p\left(s\right)\log p\left(s\right),$$

where p(s) is the occurring probability of element *s* and I(s)=−logp(s) is the corresponding self-information [28].

Various definitions exist for the probability of the occurrence of elements *s*. In this graph modeling context, graph *G* represents the discrete system S, with each node *v* corresponding to an element *s*. This study leverages the graph’s structural characteristics to redefine entropy as

(8) $$H\left(G\right)=-\sum_{v_{i}\in V}p\left(v_{i}\right)\log p\left(v_{i}\right).$$

Here

(9) $$p\left(v_{i}\right)=\frac{f(v_{i})}{\sum_{v_{j}\in V}f\left(v_{j}\right)},$$

where

(10) $$f\left(v_{i}\right)=\sum_{(v_{i},v_{j})\in E}\langle v_{i},v_{j}\rangle .$$

Thus, the entropy centrality of node $v_{i}$ is denoted as the difference between original graph and the graph without $v_{i}$,

(11) $$H\left(v_{i}\right)=H\left(G\right)-H\left(G\setminus \{v_{i}\}\right).$$

The pseudocode is shown in Algorithm 1.
**Algorithm 1:** greenGraph Entropy Function H(G)
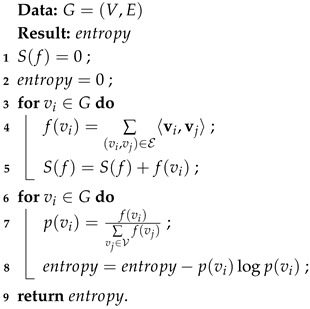


In graph neural network computations, each node in the graph obtains its representation by aggregating information from its own and its surrounding low-order or high-order neighbors. Common graph-attention-mechanism neural networks aggregate patterns [29,30,31], which adjust the weights of surrounding neighbors adaptively to obtain target node aggregation. Their basic form is

(12) $$z_{i}^{m}=\mathrm{ReLU}\left(\alpha_{i,i}\Theta_{1}X_{i}^{m}+\sum_{(v_{i},v_{j})\in E}\alpha_{i,j}\Theta_{1}X_{j}^{m}\right).$$

Here, $X_{i}^{m}\in R^{\omega }$ is the input of node (sensor) $v_{i}$, which is the *i*-th row of input $X_{i}^{m}$ in Formula (1). $\Theta_{1}$ is the weight matrix pending training, and $\alpha_{i,i},\alpha_{i,j}$ are the attention coefficients from attention mechanism.

In this work, we propose a novel attention calculation using entropy centrality. Specifically,

(13) $$\alpha_{i,j}=\frac{\exp(\pi \left(v_{i},v_{j}\right))}{\sum_{v_{r}\in \left\{v_{i}\right\}⋃\left\{v_{k}|\left(v_{i},v_{k}\right)\in E\right\}}\exp\left(\pi \left(v_{i},v_{r}\right)\right)},$$

where

(14) $$\begin{array}{cc} \pi (v_{i},v_{j}) & =\mathrm{LeakyReLU}(H\left(v_{j}\right))\\ \; & =\mathrm{LeakyReLU}(H\left(G\right)-H\left(G\setminus \{v_{j}\}\right)). \end{array}$$

### 3.3. Loss and Training

In the feature extractor layer, this work get the aggregated representations for all nodes of *m*-th sensor graph $G^{m}$, which are $Z^{m}=\left\{z_{1}^{m},\ldots ,z_{N}^{m}\right\}$. Then, this work uses the *m*-th input of overall sensor values by stacked fully-connected layers with element-wise multiply of $z_{i}^{m}$ and $v_{i}$ as input. The output is

(15) $$\begin{array}{cc} {\hat{s}}^{m} & =\{{\hat{s}}_{1}^{m},\ldots ,{\hat{s}}_{N}^{m}\}\\ \; & =f_{\Theta_{2}}\left(\left[z_{1}^{m}\circ v_{1},\ldots ,z_{N}^{m}\circ v_{N}\right]\right), \end{array}$$

where ∘ is the element-wise multiply, and $\Theta_{2}$ is the learned parameters.

This work uses the overall Mean Squared Error (MSE) as the regression loss function to evaluate the prediction on the training data. The format is

(16) $$\begin{array}{cc} L & =\frac{1}{M}\sum_{m=1}^{M}{∥{\hat{s}}^{m}-s^{m}∥}_{2}^{2}\\ \; & =\frac{1}{M}\sum_{m=1}^{M}\sqrt{\sum_{i=1}^{N}\left({\hat{s}}_{i}^{m}-s_{i}^{m}\right)}, \end{array}$$

where $s^{m}=\left\{s_{1}^{m},\ldots ,s_{N}^{m}\right\}$ is the real observed data during training.

### 3.4. Anomaly Detection

Following a malfunction in a complex system, abrupt anomalies often appear in the time-series data of its components. This paper individually computes the reconstruction through anomaly scores for each sensor to identify these anomalies. Subsequently, these sensor-specific anomaly scores are aggregated to derive an overall system anomaly score for the specified time period. For *i*-th sensor $v_{i}$, the anomaly score is

(17) $$ERROR\left(v_{i},m\right)=\left|s_{i}^{m}-{\hat{s}}_{i}^{m}\right|.$$

Given the potential for significant variability in the characteristics of different sensors, their bias values may also exhibit disparate dimensions. We implement a robust normalization procedure for each sensor’s error values to mitigate the undue influence of individual sensor biases on the overall outcome.

(18) $$NERROR\left(v_{i},m\right)=\frac{ERROR\left(v_{i},m\right)-{\tilde{\mu }}_{i}}{{\tilde{\sigma }}_{i}}.$$

Here, ${\tilde{\mu }}_{i}$ and ${\tilde{\sigma }}_{i}$ are the mean and standard deviations of $ERROR(v_{i},m)$.

After that, this work identifies the sensor with the largest anomaly score for the *m*-th input as the signal of anomalousness. This is because only a few sensors affect the overall anomalousness of the entire industrial complex system. The overall Anomaly Detection Score is

(19) $$MERROR\left(m\right)=\max_{v_{i}\in G}NERROR\left(v_{i},m\right).$$

Consequently, by setting an appropriate threshold η, MERROR(m) values exceeding this threshold can be classified as anomalies, which satisfy MERROR(m)≥η. The most straightforward approach involves utilizing the expected value derived from the training set’s statistical distribution, which represents the typical behavior in the absence of anomalies. The anomaly detection is shown in Algorithm 2.

## 4. Experiments

This section presents a comparative analysis of the proposed methodology against several state-of-the-art models for multivariate time-series anomaly detection on the SMAP, MSL, SWaT, and WADI datasets. The experimental implementations, findings, analysis, and discussion are subsequently proposed.

### 4.1. Datasets

This paper uses four publicly available multivariate time-series anomaly detection datasets, whose details are summarized in Table 1. The details of these datasets are as follows, including the volumes of the training and test sets, their dimensions, and the anomaly rates within the datasets.

- The **Soil Moisture Active Passive (SMAP)** dataset [32] encompasses soil samples and telemetry data from NASA’s Mars Rover.
- The **Mars Science Laboratory (MSL)** dataset [33] is analogous to SMAP. MSL includes sensor and actuator data from the Mars Rover. However, the dataset is distinguished by a high prevalence of trivial sequences.
- The **Secure Water Treatment (SWaT)** dataset [34] is derived from a real-world water treatment plant, comprising 7 days of normal operation and 4 days of abnormal operation. It incorporates sensor readings (e.g., water level, flow rate) and actuator commands (e.g., valve and pump control).
- The **Water Distribution (WADI)** dataset [35] is an extension of the SWaT system. The WADI dataset integrates more than twice the number of sensors and actuators compared to the SWaT model. The dataset spans a longer period, with 14 days of normal operation and 2 days of attack scenarios.

**Algorithm 2:** greenAnomaly Detection

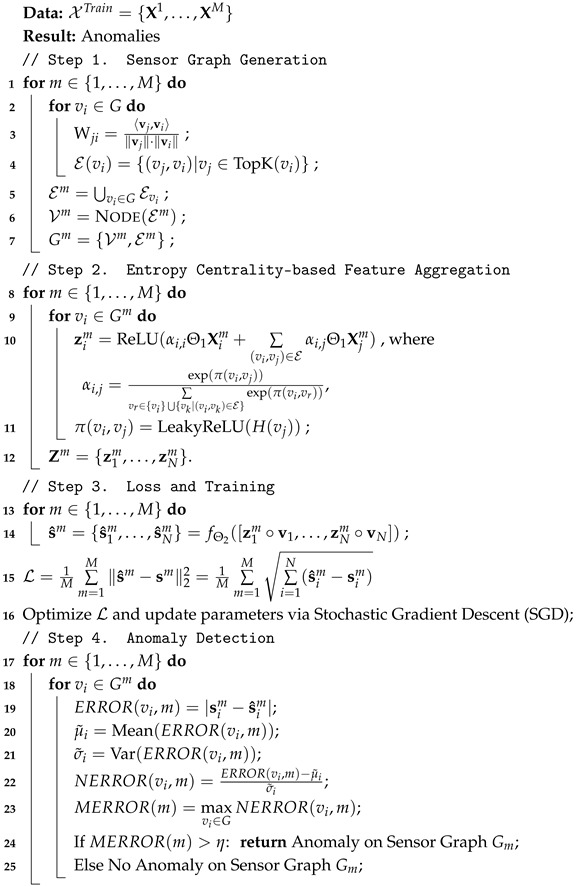



### 4.2. Baselines

This study implements twelve baseline methodologies: IsolationForest, LSTM, LSTM-NDT, MAD-GAN, MTAD-GAT, DAGMM, GDN, OmniAnomaly, USAD, ATUAD, MSCRED, and MSAD. Then, the experimental outcomes of these baseline algorithms are compared with the results of the proposed methodology.

- **IsolationForest** [36]: IsolationForest is an unsupervised anomaly detection algorithm that focuses on isolating anomalies rather than profiling normal data. It constructs random decision trees to separate outliers, which require fewer splits due to their unique characteristics. This algorithm is efficient, exhibiting low linear time complexity, and performs well on high-dimensional datasets without requiring distance calculations.
- **LSTM** [37]: LSTM (Long Short-Term Memory) is a recurrent neural network (RNN) architecture designed to capture long-term dependencies in sequential data. It utilizes specialized memory cells with input, output, and forget gates to control information flow, thereby preventing vanishing gradients in deep learning.
- **LSTM-NDT** [38]: LSTM-NDT enhances traditional LSTM by integrating adaptive thresholding for anomaly detection. It dynamically adjusts decision boundaries in time-series data, improving robustness against noise while maintaining sensitivity to true anomalies.
- **MAD-GAN** [39]. MAD-GAN is a GAN-based multivariate time-series anomaly detection method. It employs adversarial training between a generator and a discriminator to model the normal data distribution using reconstruction error for anomaly identification. Its strength lies in capturing intricate spatiotemporal dependencies, making it suitable for applications like industrial IoT. In a typical implementation, an LSTM encoder analyzes local and global anomaly patterns within high-dimensional time-series data.
- **MTAD-GAT** [40]: MTAD-GAT detects anomalies in interdependent sensors by modeling relationships via GAT. It combines feature reconstruction and forecasting errors, excelling in industrial IoT and system monitoring.
- **DAGMM** [41]: DAGMM is an unsupervised anomaly detection model that combines deep autoencoders with Gaussian Mixture Models (GMMs). It jointly optimizes dimensionality reduction and density estimation via end-to-end training, leveraging reconstruction error and low-dimensional features to identify anomalies effectively
- **GDN** [42]: GDN is a novel GNN framework that learns sensor dependencies via attention-based graph structure learning and detects anomalies by scoring deviations from learned patterns, achieving interpretability and superior accuracy on water treatment datasets
- **OmniAnomaly** [43]: OmniAnomaly is a robust stochastic recurrent neural network for multivariate time-series anomaly detection. It learns standard patterns through stochastic variable connections and a planar normalizing flow, utilizing reconstruction probabilities to identify and interpret anomalies, achieving a state-of-the-art F1-Score.
- **USAD** [44]: USAD is an unsupervised anomaly detection framework characterized by an adversarial training architecture. USAD is capable of achieving both efficient learning and high detection accuracy.
- **ATUAD** [45]: ATUAD is an unsupervised anomaly detection model that integrates adversarial training with the Transformer architecture to amplify subtle anomalies and enhance robustness.
- **MSCRED** [46]: MSCRED leverages a multi-scale convolutional recurrent encoder–decoder architecture to achieve effective spatio-temporal modeling for end-to-end anomaly detection.
- **MSAD** [47]: MSAD refers to the multivariate time series based on multi-standard fusion, which understands the data by analyzing the data density and sample spacing.

### 4.3. Parameters

The proposed methodology was implemented using a Python 3.6 and PyTorch 1.7 environment. During the experimental phase, 20% of the training dataset was designated as the validation set, with the remaining 80% allocated for training purposes. The training set is used to train the model, while the validation set assesses the performance of the trained model. This work utilizes the Adam optimizer [48] to train our model, employing an initial learning rate of 0.01 and a step scheduler with a step size of 0.5. The number of layers in the Transformer encoders is two, the number of layers in the feed-forward unit of the encoders is two, there are 64 hidden units in the encoder layers, and the dropout rate is 0.1. The most important parameters are the sliding window *w* and the sliding step *m*, which are crucial for extracting dynamic and localized information. The values used in the experiments for these two parameters are covered in Table 2.

### 4.4. Data Preprocessing and Evaluation Metrics

#### 4.4.1. Data Preprocessing

Data standardization was used before training to improve the robustness of all models. Data standardization balances and resizes each data point *x* to the maximum and minimum of the training data.

(20) $$\bar{x}=\frac{x-\min(X)}{\max(X)-\min(X)}.$$

#### 4.4.2. Evaluation Metrics

The detection performance of all models was evaluated using Precision, Recall, and F1-Score [49], in which

(21) $$\begin{array}{cc} \; & \mathrm{Precision}=\frac{\mathrm{TP}}{\mathrm{TP}+\mathrm{FP}};\\ \; & \mathrm{Recall}=\frac{\mathrm{TP}}{\mathrm{TP}+\mathrm{FN}};\\ \; & F1=2\times \frac{\mathrm{Precision}\times \mathrm{Recall}}{\mathrm{Precision}+\mathrm{Recall}}. \end{array}$$

In this context, TP denotes the true positives, which are the anomalies that are correctly identified. FP signifies the false positives, representing instances incorrectly flagged as anomalies. TN refers to the true negatives, indicating the accurate classification of normal samples. Finally, FN represents the false negatives, which are the normal samples that were incorrectly classified as anomalies.

### 4.5. Results

The anomaly detection results, including Precision, Recall, and F1-Score, for the proposed method and baseline models are presented in Table 3. Specifically, the results for the baselines are sourced from the published literature.

For the SMAP dataset, regarding Precision, the highest score of 0.8941 is achieved by LSTM, while the proposed method scores 0.8552, ranking third after LSTM and DAGMM, whose results are 0.8941 and 0.8645, respectively. Regarding Recall, the proposed method scored 0.9790, ranking third behind the top scores of 0.9991 by MTAD-GAT and 0.9891 by GDN. For the composite F1-Score, the proposed method achieves 0.9071, ranking the top. This shows that the proposed method achieves a favorable balance between Precision and Recall. For the MSL dataset, the proposed method still achieves a balanced performance. Concerning Precision, the highest score of 0.9308 was obtained by GDN, with the proposed method scoring 0.9182, ranking second, 0.0126 lower than GDN. Regarding Recall, the highest score of 1.0000 was achieved by LSTM-NDT, while the proposed method scored 0.9941, ranking second. For the composite F1-Score, the proposed method achieved 0.9546, ranking second and slightly below GDN’s 0.9591. For the SWaT dataset, the proposed method shows the best Precision over all baselines. In terms of Precision, the proposed method achieves the highest score of 0.99828, which is 0.0046 higher than the second result, achieved by OmniAnomaly. For Recall, the highest score is achieved by MAD-GAN at 0.6957, while the proposed method scores 0.6563, which is 0.0394 lower than MAD-GAN. For the composite F1-Score, the proposed method achieves 0.7870. For the WADI dataset, concerning Precision, the highest score is achieved by OmniAnomaly at 0.3158, with the proposed method scoring 0.3029, which is 0.0129 lower than OmniAnomaly. Regarding Recall, the proposed method scored 0.8196, lower than DAGMM’s 0.9981 and MAD-GAN’s 0.9124. For the F1-Score, the proposed method achieved a score of 0.4423, ranking first and outperforming the second-place GDN and OmniAnomaly at 0.4260.

In summary, the proposed method performs favorably on the SMAP, MSL, and WADI datasets. On these datasets, the proposed method ranks first or second overall in terms of the F1-Score. On SWaT, the proposed method fails to yield a favorable result in terms of the overall F1-Score, but demonstrates a powerful discrimination ability regarding Precision.

### 4.6. Ablation Experiment

The primary innovation of this paper lies in the complex network association of sensors, coupled with node feature aggregation via graph neural network computation, leveraging a method of entropy analysis for complex systems. This section removes the influence of Entropy Centrality-based Feature Aggregation, where in Formula (12), $\alpha_{i,i}$ and $\alpha_{i,j}$ are assigned equal weights, i.e., $\alpha_{i,i}=\alpha_{i,j}$.

(22) $$\left\{\begin{array}{c} Entropy\;Centrality:\;z_{i}^{m}=\mathrm{ReLU}\left(\alpha_{i,i}\Theta_{1}X_{i}^{m}+\sum_{(v_{i},v_{j})\in E}\alpha_{i,j}\Theta_{1}X_{j}^{m}\right),\\ Remove\;Entropy\;Centrality:\;z_{i}^{m}=\mathrm{ReLU}\left(\Theta_{1}X_{i}^{m}+\sum_{(v_{i},v_{j})\in E}\Theta_{1}X_{j}^{m}\right). \end{array}\right.$$

**Figure 5:**
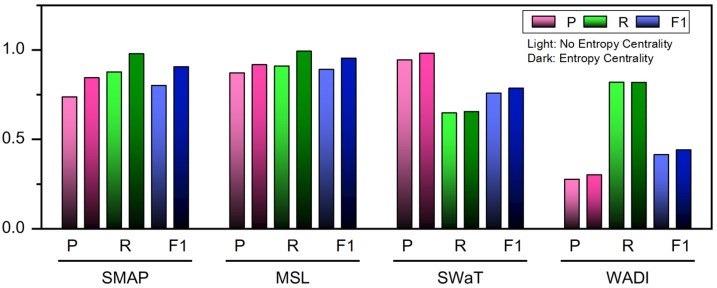
Ablation experiments for entropy centrality (P for Precision, R for Recall, and F1 for F1-Score).

Ablation experiments were conducted by comparing the computational results before and after removing entropy centrality to validate the effectiveness of the entropy centrality design. The compared results are shown in Figure 5 and Table 4. It is evident that the proposed method enhances performance compared to the method that removes entropy centrality.

### 4.7. Discussion

This paper employs unsupervised experiments and an ablation study to evaluate the effectiveness of the proposed method. In the unsupervised experiments, our approach demonstrates superior performance, achieving excellent results on key metrics including Precision and F1-Score. The ablation study further validates the core innovation of this work: the entropy-centric attention mechanism. Unlike the ablation baseline, which fuses data using neural networks alone and treats all neighboring nodes equally, our proposed mechanism assigns differentiated weights to high-order neighboring nodes based on their topological positions. The results conclusively show that this entropy-based mechanism is vital for the effective aggregation of node features and constitutes the principal advantage of our method over other network-based approaches.

Regarding benchmarks of graph-based methods, existing approaches often rely on pre-defined or learned graph structures, such as using correlation or proximity measures, to model relationships among time-series sensors. While these methods incorporate graph neural networks for information aggregation, they typically treat neighboring nodes uniformly or assign attention weights based on superficial node feature similarities. In contrast, our method introduces an entropy-based method by quantifying the informational importance of nodes through entropy centrality. This allows the model to assign differentiated attention weights not merely based on features, but on the relative uncertainty and influence of a node within the higher-order neighborhood structure. Consequently, our mechanism can effectively capture connection in sensor networks, which is often critical for identifying anomalies that are missed by methods using static or feature-only attention.

The proposed model consists of four main components, which are Sensor Graph Generation, Entropy Centrality-based Feature Aggregation, Loss and Training, and anomaly detection. Among the four modules, the core module is Entropy Centrality-based Feature Aggregation. The advantage of this method lies in optimizing the attention module solely through entropy centrality, which can be generalized to other attention-based methods. For an *n*-node graph, the complexity of Sensor Graph Generation is $O(d\cdot n^{2}+logK\cdot n^{2})$, where d denotes the dimension of the feature vector of each node $v_{i}$. The complexity of calculating entropy centrality is $O(n\cdot |E|\cdot d+n^{2})$; the complexity of Feature Aggregation is $O(n\cdot d_{in}\cdot d_{out}+n^{2})$. The complexity of Loss and Training is O(m·n). The complexity of anomaly detection is O(n). Thus, the overall upper bound of the proposed model is $O(n^{2})$.

**Figure 6:**
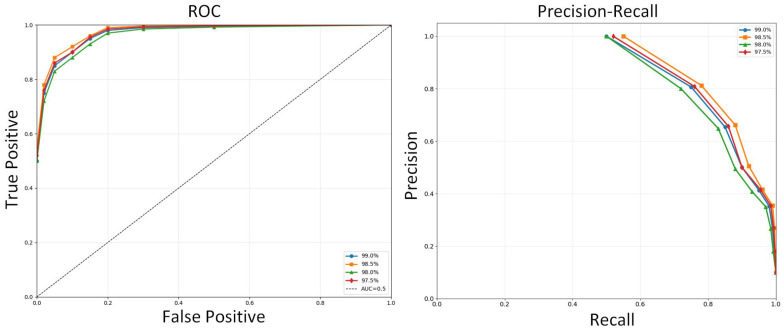
ROC and AUC curves based on different proportions of anomalous data in the MSL dataset.

The hyperparameter that influences anomaly detection is threshold η. As for the selection of threshold η, we used the common “defining anomalies by proportion” method [50]. This method defines the threshold by the proportion of the data the work is willing to label as anomalous. For instance, in this manuscript, we assume each dataset is expected to contain 1% anomalies, so the system will rank all anomaly scores in ascending order and demarcate a cutoff point immediately following the top 1%. All data points with scores exceeding this threshold will be classified as anomalies. Figure 6 shows the ROC and AUC curves based on different proportions of anomalous data in the MSL dataset.

## 5. Conclusions

In various industrial scenarios, researchers diagnose anomalies within industrial systems by analysing time-series data recorded by sensors. Specifically, sensors interact and correlate to form complex systems, the structure of which is determined by the characteristics of the sensors, such as sensor feature data and recorded data. This paper utilizes a graph-based network model to model the implicit structure within complex industrial systems. Further, it represents and analyzes the graph structure through graph neural networks for multivariate time series. Addressing the information aggregation representation problem of graph nodes in neural network calculations, this paper proposes an entropy-based attention mechanism to induce the aggregation and representation of information from neighboring nodes to target nodes. The advantage of the proposed method lies in its ability to mine the implicit interaction relationships between multivariate time series from the perspective of complex system structure modeling and to determine the implicit anomalous characteristics in multivariate time series from the perspective of complex system graph structure analysis.

Several directions warrant further discussion in future work. One important area is the problem of effective anomaly detection under small-sample conditions. Industrial scenarios often have only a small amount of labeled data; therefore, unsupervised, semi-supervised, and few-shot learning have greater applicability. Specifically, effectively augmenting a small number of labels or anomalous samples is necessary to ensure the model’s effectiveness. Another related point of interest involves the window length and sliding distance hyperparameters for time-series training-sample sampling, which are often optimized through multiple experiments. These parameters often determine the amount of information contained in the data used for training. Therefore, analyzing the quantitative relationship between these hyperparameters and the amount of information is of great significance for quickly estimating key hyperparameters under new datasets.

## Figures and Tables

**Table 1:** The details of four publicly available multivariate time-series anomaly detection datasets (SMAP, MSL, SWaT, and WADI).

Datasets	Train	Test	Dimensions	Anomaly Rate
**SMAP** [32]	135,183	427,617	25 (55)	13.13%
**MSL** [33]	58,317	73,729	55 (3)	10.72%
**SWaT** [34]	496,800	449,919	51 (1)	11.98%
**WADI** [35]	1,048,571	172,801	123 (1)	5.99%

**Table 2:** Hyperparameters (sliding window *w* and sliding step *m*) used in different datasets.

Datasets	Sliding Window *w*	Sliding Step *m*
**SMAP**	120	10
**MSL**	95	10
**SWaT**	50	10
**WADI**	135	10

**Table 3:** Quantitative results for the proposed method and baselines on four real-world datasets. A higher value indicates a better performance for these three metrics (P for Precision, R for Recall, and F1 for F1-Score). For the results of this work, the upper-right number indicates the ranking within the column.

Method	SMAP			MSL		
	P	R	F1	P	R	F1
IsolationForest [36]	0.5239	0.5907	0.5553	0.5394	0.8654	0.6645
LSTM [37]	**0.8941**	0.7813	0.8339	0.8545	0.8250	0.8359
LSTM-NDT [38]	0.8523	0.7326	0.7879	0.6288	**1.0000**	0.7721
MAD-GAN [39]	0.8157	0.9216	0.8654	0.8516	0.9930	0.9169
MTAD-GAT [40]	0.7991	**0.9991**	0.8880	0.7917	0.9824	0.8768
DAGMM [41]	0.8645	0.5673	0.6851	0.8960	0.6393	0.7462
GDN [42]	0.7480	0.9891	0.8518	**0.9308**	0.9892	**0.9591**
OmniAnomaly [43]	0.8130	0.9419	0.8728	0.7848	0.9924	0.8765
USAD [44]	0.7480	0.9627	0.8419	0.8710	0.9536	0.9104
ATUAD [45]	0.8157	0.9999	0.8985	0.9041	0.9999	0.9496
MSCRED [46]	0.8175	0.9216	0.8664	0.8912	0.9862	0.9363
MSAD [47]	0.8502	0.9066	0.8775	0.9065	0.9626	0.9337
This Work	${0.8452\;}^{3}$	${0.9790\;}^{3}$	${0.9071\;}^{1}$	${0.9182\;}^{2}$	${0.9941\;}^{2}$	${0.9546\;}^{2}$
**Method**	**SWaT**			**WADI**		
	**P**	**R**	**F1**	**P**	**R**	**F1**
IsolationForest [36]	0.4929	0.4495	0.4702	0.1822	0.7824	0.2954
LSTM [37]	0.8615	0.8327	**0.8469**	0.2063	0.7632	0.3358
LSTM-NDT [38]	0.7778	0.5109	0.6167	0.2138	0.7823	0.3358
MAD-GAN [39]	0.9593	**0.6957**	0.8065	0.2233	0.9124	0.3588
MTAD-GAT [40]	0.9718	0.6957	0.8109	0.2818	0.8012	0.4169
DAGMM [41]	0.8992	0.5784	0.7040	0.2760	**0.9981**	0.4324
GDN [42]	0.9697	0.6957	0.8101	0.2912	0.7931	0.4260
OmniAnomaly [43]	0.9782	0.6957	0.8131	**0.3158**	0.6541	0.4260
USAD [44]	0.9977	0.6879	0.8143	0.1873	0.8296	0.3056
ATUAD [45]	0.9696	0.6957	0.8101	0.3005	0.8286	0.4403
MSCRED [46]	0.9318	0.9825	0.9565	0.2513	0.7319	0.3741
MSAD [47]	0.9532	0.9943	0.9739	0.2832	0.8356	0.4230
This Work	${0.9828\;}^{1}$	${0.6563\;}^{6}$	${0.7870\;}^{6}$	${0.3029\;}^{2}$	${0.8196\;}^{3}$	${0.4423\;}^{1}$

**Table 4:** Ablation experiments for entropy centrality (P for Precision, R for Recall, and F1 for F1-Score).

Method	SMAP			MSL		
	P	R	F1	P	R	F1
Entropy Centrality	0.8452	0.9790	0.9071	0.9182	0.9941	0.9546
Without Entropy Centrality	0.7386	0.8775	0.8021	0.8725	0.9112	0.89143
**Method**	**SWaT**			**WADI**		
	**P**	**R**	**F1**	**P**	**R**	**F1**
Entropy Centrality	0.9828	0.6563	0.7870	0.3029	0.8196	0.4423
Without Entropy Centrality	0.945	0.6489	0.7642	0.2781	0.8200	0.4152

## Data Availability

The data presented in this study are available on reasonable request from the corresponding author.
